# Organizational Structure Change and Hybridity: Enhancing Uncertainty as a Response to Competing and Changing Institutional Logics

**DOI:** 10.3389/fpsyg.2022.854319

**Published:** 2022-03-09

**Authors:** Liming Liu, Chao Zhang

**Affiliations:** School of Communication and Design, Sun Yat-sen University, Guangzhou, China

**Keywords:** organizational structure, hybridity, uncertainty, China, mass media organizations

## Abstract

Confronting the uncertain environment, this article adopts a case research approach to resonate with the studies of hybridity. It aims to explain how the perception of uncertainty in the institutional environment affects the adaptation of organizational structure in pursuing legitimacy for hybrid organizations. Based on the empirical data collected from a two-staged fieldwork and in-depth interviews, the case analysis concentrates on the correlation between the evolution of institutional logics and organizational structure change from a diachronic perspective. The findings indicate that in the face of competing and changing institutional logics, Chinese mass media organizations have gradually shifted from a dominated blending strategy in the exploration stage to a deeply compartmentalizing strategy in the stable stage. The hybrids can deal with the uncertainty of the institutional environment by enhancing the uncertainty of the organizational structure. Consequently, the case evolves an organizational integration through internal legitimacy. It manifests a possibility for hybrids of combining the two major response mechanisms in one process.

## Introduction

Digital media and mobile communication technology are bringing great challenges to mass media organizations. Scholars have conceptualized organizational change differently to describe this global change, such as “liquid journalism” (Deuze, 2008), “uncertain times” (Lowrey and Woo, 2010), “blurring boundaries” (Loosen, 2015), and among others. These crisis discourse altogether point to a core theme: how the news media would find a new development path in an extremely uncertain environment. However, the reality has shown that Chinese mass media organizations have not found a feasible solution to solve the technical and commercial difficulties in the changing market even after a long time of exploration.

According to the theory of organizational sociology, when the organizational objectives as a measure of organizational performance or achievements are ambiguous, organizations tend to resort to a legitimacy mechanism for their viability (Zhou, 2003, pp.89). This trend is also evident in the media sector. Most relevant studies explored the impact of a specific institutional framework on organizational behaviors. These included the intervention of political power (Dickinson and Memon, 2012; Usher et al., 2021), performance legitimacy in the era of economic crisis (Simon and Graves, 2019), social expectation of professional news production (Carlson, 2017; Broersma and Singer, 2020), and upgrade of media technology (Tandoc and Maitra, 2018; Liu and Berkowitz, 2020). However, different institutional elements could not function on its own in China’s social context. They often exist at the same time, conflict with each other, and continue to evolve (e.g., Wei, 2019).

Therefore, the change of Chinese mass media organizations provides valuable experience for understanding how organizations respond to the uncertain institutional environments. Although some available literature treats organizational change as a continuous process (Burnes, 2004; Zhang et al., 2015), “organizational structure” has been regarded as an apparent indicator and clear evidence of this discrete field (Král and Králová, 2016). There are two research approaches explaining the process of the organizational structure change. The traditional theory emphasizes the coercive impacts of institutions on the organizational structure with a consequence of isomorphism (DiMaggio and Powell, 1983). But recent studies suggest that a favorable way for organizations to maintain or regain legitimacy in the heterogeneous institutional environment may be to combine different and potentially contradictory legitimation strategies (Scherer et al., 2013; Schembera and Scherer, 2017). As a result, a sort of “hybrid organization” would be formed as a combination of different institutional logics embedded in the organization (Battilana and Dorado, 2010; Battilana et al., 2015).

Essentially, mass media constitutes the typical hybrid organization. Therefore, the purpose of this article is actually to answer how the perception of uncertainty in the institutional environment affects the adaptation of organizational structure in pursuing legitimacy for hybrids. This article begins with a brief overview of the academic context and core issues of hybridity. Their discussions of the competing process of institutional logics are beneficial. However, the previous literature has not paid enough attention to the changes of institutional logics themselves, thus lacking sufficient response to the complexity of the institutional environment. In an effort to fill this gap, this article introduces a new variable of “institutional change” guided by the perspective of institution-as-process (Thelisson and Meier, 2020). Together with “institutional competition,” it constitutes two dimensions of “institutional uncertainty.” On this basis, a new theoretical framework has been constructed to analyze the interactive relationship between organizational behavior and the institutional environment. There are two major responding mechanisms of hybridity to different institutional logics—“blending” and “compartmentalizing” (Beaton et al., 2021).

The empirical data is collected from a two-staged fieldwork and semi-structured interviews in a Chinese metropolis daily between 2016 and 2021. In the case analysis, this article divides the evolution of the critical case into three stages over time: the exploration stage, the conflict stage, and the stable stage. It indicates that in the face of competing and changing institutional logics, Chinese mass media organizations have gradually shifted from a dominated blending strategy in the exploration stage to a deeply compartmentalizing strategy in the stable stage. They dealt with the uncertainty of the institutional environment by enhancing the uncertainty of organizational structure. Consequently, an internal legitimacy is achieved through interaction within the organization to ease internal tensions, thereby promoting an organic combination of the two strategies. These findings not only contribute a better understanding of hybrid organizations’ managerial strategic choices facilitating their sustainability, but also respond to the similarity and variance of newspaper crises around the world (Siles and Boczkowski, 2012).

## Hybridity: Organizational Structure in the Uncertain Institutional Environment

The organization studies have proposed that organizations would face two different uncertainties of the environment: objective uncertainty and perceptive uncertainty (Downey et al., 1975). From a microscopic perspective, a variety of studies have explored the impacts of perception of environmental uncertainty on organizational behavior. For example, organizational structures (Lawrence and Lorsch, 1967; Duncan, 1973), organizational strategies (Christine, 1991), political processes within organizations (Child, 1972), and the organizational fields (Vermeulen et al., 2016). Following this path, the article focuses on the uncertainty of the institutional environment and discusses how the perception of institutional uncertainty in organizational change affects the adaptation of organizational structure.

Organizational structures in the uncertain institutional environments can be placed in the theoretical spectrum of “hybridity”^1^ (Minkoff, 2002; Battilana and Dorado, 2010; Pache and Santos, 2013). Some other scholarly camps distinguish sectors, societal domains (Brandsen et al., 2005; Minkoff et al., 2008; Billis, 2010), or organizational identities (Glynn, 2000; Pratt and Foreman, 2000). The camp taking institutional stance views hybridity as the combination of pluralistic institutional logics (Skelcher and Smith, 2015; Wells and Anasti, 2019). This approach is prominent in the publicity-oriented organizational fields like social enterprises (Pache and Santos, 2013; Wry and York, 2017) and non-profit organizations (Battilana and Dorado, 2010; Zhang, 2017; Beaton et al., 2021). Likewise, mass media essentially combines various values such as economic performance, political participation, and public communication, thus constituting a typical hybrid organizational structure.

Because of the latent contradictions caused by competitions for resources and legitimacy among different institutional elements (Oliver, 1991), how to handle and manage the conflicts between disparate logics has become the central issue in the study of hybridity (Kraatz and Block, 2008; Pache and Santos, 2013; Battilana et al., 2015). Scholars believe that to reduce these tensions, it is necessary for organizations to construct adaptive structures based on the specific context (Greenwood et al., 2011; Fitzgerald and Shepherd, 2018). Relevant literature has detailed analyzed a variety of structural forms including coalition, out-sourced firm, subsidiary corporation, and others (Smith, 2010; Beaton et al., 2021). Therefore, the adaptation of organizational structure can be used as a significant mediator to illustrate hybridity in response to different institutional logics.

However, previous studies concentrate more on the competitive nature of the institutional environment and the tensions it creates. The dynamic process of organizational structure change has been ignored (Smith and Besharov, 2019). As stated by Pache and Santos (2013), “Understanding the dynamic process through which organizational responses shape organizational structure, which in turn influences subsequent responses, is an important next step in uncovering the complexity of institutional process.” Although some scholars have noticed organizations’ ongoing adaptive enactment process (Jay, 2013; Dalpiaz et al., 2016; Smith and Besharov, 2019), their findings mainly revealed how the paradoxical frame of hybrids influences the flexibility and stability of the organizational structure. The adaptation caused by the change of institutional logics themselves has still not been fully explained which is exactly the crucial context of this article.

Some scholars highlight that legitimacy is an ambiguous concept and the legitimation dynamics need more in-depth investigation (Suddaby et al., 2017; Thelisson and Meier, 2020). In this respect, they defined legitimation as a non-linear process inherently contested and negotiated in everyday activities in relation to organizational actions and decisions (Gnes and Vermeulen, 2019; Thelisson and Meier, 2020). For example, Thelisson et al. (2018) have explained the evolution of intertwined institutional logics in the merger integration and the relative balance between the logics in play from a managerial perspective. However, this research major concerns the way certain institutional logics coexist and how their relationship evolves in organizational or inter-organizational change. The re-conceptualization of “institutional uncertainty” in this article would provide new empirical evidence for understanding this process deeply.

## Competition and Change: Institutional Logics of Mass Media Organizations

Institutional logics affect organizational behaviors through legitimacy mechanisms. Legitimacy is defined here as “a generalized perception or assumption that the actions of an entity are desirable, proper, or appropriate within some socially constructed system of norms, values, beliefs, and definitions” (Suchman, 1995) or it measures the degree to which actors are accepted or supported by stakeholders (Zimmerman and Zeitz, 2002). Organizational legitimacy has different classification standards because of the differences in institutional sources. Scott (2010) divided legitimacy into three categories: regulative legitimacy, normative legitimacy, and cultural-cognitive legitimacy. In addition, some scholars complemented social benefit legitimacy or practical legitimacy according to the interest orientation of enterprise organizations, such as industrial legitimacy (Zimmerman and Zeitz, 2002) and market legitimacy (Dacin et al., 2007). According to these definitions, the sources of legitimacy, in other words, the institutional logics of mass media organizations are shown in Table 1.

**TABLE 1:** Multiple legitimacy of mass media organizations.

Types of legitimacy	Institutional connotation	External representations
Regulative legitimacy	Government-issued laws and regulations regarding news media	Guidance on media convergence, governance, etc.
Normative legitimacy	professional identification with news values by public and stakeholders	Professionalism, Objectiveness, etc.
Cultural-cognitive legitimacy	Public and stakeholders’ expectations of news meanings; cultural perceptions related to “news”	Public nature of news, guidance by correct values, Ideology of the Party, etc.
Social benefit legitimacy	Whether the news media conforms to the overall judgment of technical environment, economic benefits and public interests	Technical innovation, media influence, business innovation, etc.

Table 1 describes the institutional pluralism for mass media organizations. On this basis, the conceptualization of “institutional uncertainty” in this article contains two core variables based on the previous studies: “competitiveness” and “change.” Both manifest the dynamic and time-varying nature of institutional logics in line with the process analysis of this article.

Institutional competition refers to the extent to which the institutional logics is incompatible and whether there is a settled or widely accepted prioritization of the logics within the field (Raynard, 2016). It is the basic theoretical premise in the studies of hybridity. As institutional uncertainty is characterized by the multiple, competing, and sometimes conflicting institutional logics (Greenwood et al., 2011; Pache and Santos, 2013). Many scholars have noticed this complexity of the Chinese media institutional environment. For example, while being challenged by the digital media, political power has enhanced its ability to control the media (Chen and Zhang, 2019). Meanwhile, Journalism is deeply affected by “commercialism” (Li and Chen, 2016), as well as calling for the return of public responsibility at the social level (Pan and Lu, 2017).

Institutional change is measured by the freedom of evaluation criterion of specific institutional logic. High-level freedom implies a lack of explicit judgment about whether the organization is legitimate (Lin et al., 2017). The institutional ambiguity exacerbates the risk of high uncertainty of regaining legitimacy to implicate organizational transformation for survival and sustainability. This is especially true in the Chinese political-economic environment after the reform and opening-up policy (e.g., Chen et al., 2016) and in the Chinese media context with digital technology (e.g., Li, 2017). Furthermore, some studies reveal that the evolution of Chinese media logics gradually generates two dimensions of “institutional change.”

On one hand, the internet has been changing the deep structure and overall ecology of Chinese journalism from the industrial structure, regulatory system to the production process (Zhang and Wu, 2016). Consequently, as Pan and Lu (2017) mentioned, a series of questions become openly pending such as “How should news be done?” and “What norms should it adhere to?” News production takes on a liquid character (Lu and Zhou, 2016). On the other hand, the core political, economic, and social expectations for Chinese mass media organizations are suffering a loss of consensus. For instance, the political pressure on mass media transforms into a broad demand of “New mainstream media” based on the traditional censorship. The business innovation is also in face of some disputes of “continuous innovation” or “disruptive innovation” (Zeng and Wang, 2019). Additionally, both of them form a new balance which is increasing the uncertainty of the institutional environment.

## Blending or Compartmentalizing: Response Mechanisms of Hybridity

According to the hybridity literature, legitimation is a complicated decision-making process. Besharov and Smith (2014) proposed a new analysis framework for understanding organizations’ strategic choices by combing the degree of centrality and the degree of incompatibility. More specifically, Beaton et al. (2021) classified the responses of hybrids to tensions through adaptation of organizational structures into three broad categories: denying, compartmentalizing, and blending. Denying means the hybrid might eschew hybridity altogether which results in the maintenance of a single organizational form linked to the dominant logic (Beaton et al., 2021). This strategy in some studies has been described as an important solution for organizations to navigate complicated institutional terrain (Uzo and Mair, 2014). But it is not consistent with the practical experience of mass media organizations, so this article adopts the latter two mechanisms.

Blending refers to the integration of competing institutional logics within the organization through a common identity to form a unified legitimacy in which various institutional logics reinforce each other (Beaton et al., 2021). The core notion of this strategy indicates the blurring boundary between different logics (Murray, 2010). In a notable example, Battilana and Dorado (2010) compared two micro-finance organizations. They found that new hybrids can strike a delicate balance between different logics by creating a common organizational identity *via* hiring strategies and the socialization process. This strategy is also adopted by Chinese mass media organizations that absorbed the market-oriented Metropolis during the integration of enterprise conglomeration (Zhao, 2000). However, the emerging dominant logic is not a global one over the others, but instead requires to be expressed in the stages of evolution (Thelisson et al., 2018).

Compartmentalizing emphasizes the coexistence of institutional logics and suggests that hybrids can entail isolating logics in different organizational departments, divisions, or subsidiaries (Fitzgerald and Shepherd, 2018; Beaton et al., 2021). In discussing how organizations manage multiple identities related to different institutions, Pratt and Foreman (2000) defined “compartmentalizing” as an important response mechanism. It means that the organizational members choose to retain all current identities without seeking synergy within the organization. A few research literatures has explained the organizational structure change featured with the segregation between “news gathering sector” and “marketing sector” in the market-oriented reform of mass media (e.g., Li and Fang, 2010). While this strategy helps increase the flexibility of hybrids, it can also create new conflicts because different logics would guide organizational decisions and behavior simultaneously.

## Data and Methods

This article employs a qualitative research design based on a single case study under a diachronic perspective with the data collected from a two-staged fieldwork and twenty-three semi-structured interviews. The critical case is a metropolis daily (hereafter as “N”) in a China’s province (hereafter as “G”). N is the most important market-oriented newspaper in the provincial newspaper group of G’s and it is also the industry benchmark of Chinese mass media. Since 2012, N has experienced nearly a decade of reform and exploration, which makes it significantly representative theoretically and empirically. In terms of the data analysis, this article opts for a deductive method guided by a new theoretical framework. First, this article re-conceptualized the term of “institutional uncertainty” and introduced two core responding mechanisms of hybrids based on previous research. The case analysis concentrates on the correlation between the evolution of institutions and organizational structure change in three stages.

Data collection was mainly completed through a two-staged fieldwork. In the early stage, from October 2016 to January 2017, the authors got a rough idea of the reform strategies and N’s overall organizational structure framework. Then in the later stage, from June to September 2017, the authors had collected many first-hand materials aimed at organizational structure including internal documents, work-flow information, and communication among insiders. During the fieldwork, one of the authors conducted the investigation in the news headline department of *N* as an intern editor for 8 months. Her full participation in the routine work collected the direct data of the interaction inside and outside the organization.

Additionally, the authors conducted semi-structured interviews with the currently active or resigned staff of *N* at different levels from 2016 to 2021 because this period covered the whole process of *N*’ structural change from the initial exploration to a relative stable condition. The twenty-three interviewees consisted of two parts: fourteen journalists, editors, and department leaders acquired through snowballing during the fieldwork, and nine added interviewees between 2020 and 2021. These new interviews aimed to supplement data on the new organizational practice of N after 2017. Multiple repeat interviews with a few interviewees were conducted for comparative arguments about the organizational change in different stages. The interviews were conducted face-to-face lasting about 2 h and semi-structured concentrating on the core issues with some free discussion. All the interviews were audio-recorded with permission and given pseudonyms for the protection of their identities.

## Case Analysis

The case analysis indicates that enhancing the uncertainty of organizational structure can help mass media organizations better manage the hybridity by creating a flexible adaptive space. In this section, the evolution of N has been divided into three stages over time with the purpose of uncovering the response mechanisms of mass media organizations in different stages.

### Exploration Stage: The Blending Strategies of Hybridity Dominated by Technical Legitimacy

#### Adaptation of Hybridity

The main task of Chinese mass media is to meet the growing information demand through news supply. Therefore, the organizational structure in the early days was set around the allocation of news production resources and market signals, manifested as a typical bureaucratic model based on efficiency mechanism. It was decomposed from top to bottom. The editorial committee was fully responsible for detecting the public opinion environment and arranging the reporting tasks of news departments. At the horizontal level, each department consisted of a specific team of editors and journalists who completed their own tasks independently.

**FIGURE 1:**
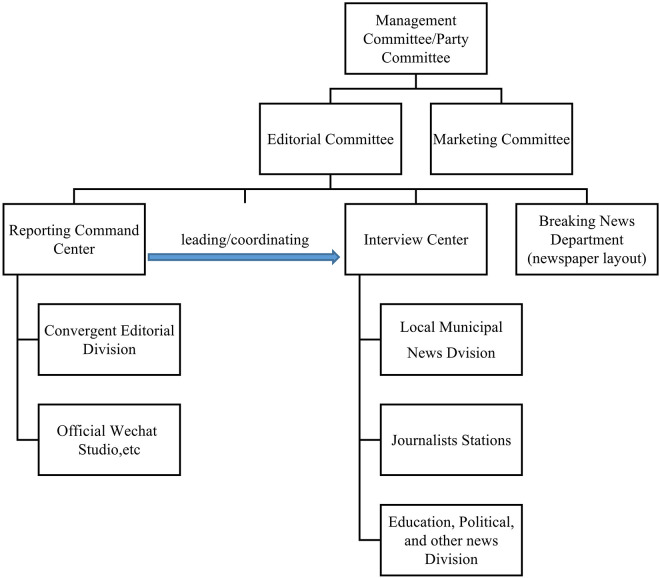
The organizational structure of N in the exploration stage.

However, the prevalence of new media delegitimized the traditional organizational structure. Adapting to the new production modes and communication channels had become the primary mission of mass media organizations in the exploration stage. N began to build a “central kitchen” in 2016 by adding a new decision-making body to the original functional hierarchy which was named “the reporting command center.” The new organizational structure is demonstrated in Figure 1. This reform merged the editorial staff originally scattered in various news departments into a large editorial department. It undertook the integration and coordination between the editorial committee and the interview departments. Its primary mission was planning news topics and publishing real-time news information. The editors were required to liaise more with reporters and arrange their reporting tasks according to the breaking news. Therefore, when newspaper layouts had been extremely compressed, the news management authority was centralized to the reporting command center.

To solve the insufficient content supply and the deteriorating business crisis, N added two complementary institutional modes into the new organizational structure: “contract-based system” and “project-based system.” Except a few divisions, N promoted the contracted “platform” reform in the Interview Center. Each “platform” signed an agreement about the business target with the organization on an annual basis. After the year-end revenues were turned over, the balance can be distributed internally. The leader of the “platform” determined how the specific year-end bonus should be distributed. Meanwhile, the project-based system included an independent project team and editor-led virtual studio for creating high-quality news columns and expanding brand influence. The specific arrangement was subjected to the operation of news content. For example, some special topics or dynamic content required the operation of specific journalists, thereby the team is more changeable^2^.

Concerning the principal-agent relationship within this organizational structure, the human resource management of N still adopted the traditional “piecework” salary assessment system. It followed the basic principle of more pay for more work and took the quality of news manuscripts as the main evaluation standard. The performance evaluation of journalists and editors was the responsibility of department heads. The performance evaluation process of a typical journalist was as follows:

First, there will be a fixed basic salary, which is generally low. Then, each manuscript mainly undergoes two steps: preliminary review and final review.

During the preliminary review, the department heads will grade the manuscript, such as excellent, medium, and poor. At the final review, the system will do a mathematical processing of the grade and the number of words of the manuscript. The newspaper office has its own calculation formula^3^.

Furthermore, the organization had a unified cap for each department regarding the total amount of salary assessment. But department heads also had limited right to pay more wages than the set amount. Consequently, on the premise of strengthening news censorship and human capital management, the media organization had given news departments some autonomy which was even further expanded after the “platform” reform.

#### Legitimacy Correlation

The organizational structure adaptation of N in the exploration stage took a dominating blending strategy. Although this media organization had enhanced its internal autonomy by adopting a flexible project-based system and strengthening the market decision-making power of different news departments, the decision-making power of news production had been centralized to the reporting command center. As a result, the reporters had to undertake multiple tasks, and the human resource management was still centered on “news content.” Thus, it can be seen that this organizational structure actually integrated different institutional logics into a news-led organizational system. Its correlation with the institutional environment is as below:

##### Institutional Competition

Technical legitimacy dominated among the institutional logics and was consistent with political legitimacy. At the beginning, the biggest crisis of the institutional environment comes from the development of new technology. Digital media, especially the internet platforms, requires the mass media organizations to adopt new technologies and re-establish the connection with the audience. In 2014, the Chinese central government promoted the policy of deep media convergence which was regarded as a reform path in line with the competing political needs, news value, and technical innovation simultaneously.

##### Institutional Change

The logic of social benefit legitimacy changed over time. Since the steep decline of advertising revenue in 2012, Chinese mass media organizations have been continuously exploring a survival model. But skateholders (e.g., enterprises) these days expect mass media organizations to provide integrated marketing strategies in various dimensions. The market-oriented media more depends on its content production capacity, resource integration capacity, and the brand influence instead of advertisements. N’s reform of “platform” was exactly the effort to increase cost consciousness within the organization and encourage employees to actively explore new business models through new incentive mechanisms.

Change 1: To gain political and technical legitimacy, mass media organizations adopted a centralized decision-making mechanism in the form of “central kitchen,” while increasing organizational flexibility with “project-based” system.
Change 2: To gain social benefit legitimacy, mass media organizations gave news departments more market decision-making power and residual rights of control to explore new business models.
Change 3: Different institutional logics were reconciled in the organizational structure and formed an organizational integration centered on news content.

### Conflict Stage: The Internal Tensions of Hybridity and Changes of Institutional Logics

The organizational structure adaptation of N had gone through an experimenting period of about 2 years between 2016 and 2018. In view of the internal adjustment and external environment, there were unavoidable obstacles in this organizational structure.

#### Internal Tensions

In the first place, new institutional designs such as “contracted platform” and “project-based system” brought more serious problems of management differentiation. As the head of each department had their own considerations on practical situations and personal interests, whether the “central kitchen” could function well was subject to the support of department heads. After all, “when the leaders (of the organization) asked the editors to carry out news planning, only journalists interested in the topic can be summoned. Otherwise, the editors can do nothing because journalists report to their (department) heads.”^3^

For example, sometimes a leader finds a topic very interesting, but its implementation depends on whether department directors cooperate. Some directors are very strong willed. You do not expect to use his journalists. Some (directors) may be quite supportive, and (if) senior leaders are also by the side, (journalists’ cooperation) will be relatively easy. There also might be some directors who respect the journalists’ personal preferences. If the journalist is willing and does not affect the routine of his department, it does not matter^4^.

Based on this management model, journalists also began to generate additional cost-benefit trade-offs: “Journalists just want to do what they are willing to do. It takes a lot of energy to select topics, interview, and write. Especially after the newspaper layout had been reduced dramatically and many journalists had resigned, every (journalist) must deal with a large amount of work. In addition, the newspaper office, particularly the leaders required news distribution *via* mobile terminals to go viral like explosion. Journalists thus felt that it’s better to produce news that would go viral for higher income and visibility, rather than spending too much time on writing useless manuscripts.”^5^

Moreover, the “platform” reform led to a more serious risk that might disrupt the organization, like a midlevel head remarked:

Since the “platform” reform, the separation between departments would be more outrageous. As each platform bears the business task of its own, then (the question will be raised) why I should work with you or why you do not work (with me), problems will arise. If I coordinate with you, I will have to charge you. How should I calculate the money? If you do not coordinate with me, then I will be forced to recruit art editors, video personnel, and researchers by myself. In the end, each platform will have to enlarge, which is not conducive to the management of the newspaper office^6^.

#### Changes of Institutional Logics

Apart from the internal tensions, it should even more attribute the failure of the two reform strategies to the changes in institutional logics from 2016 to 2018. On the one hand, the “central kitchen” was indeed regarded as a promising transformation for a time. But it lost legitimacy after a period of experimentation as its nature of centralization is contrary to the decentralization of the internet. In other words, the public awareness of technical innovation for mass media organizations became more and more obscure. On the other hand, most platforms of N cannot fulfill their business tasks signed with the organization. “Because there is a great contradiction in forcing content creating journalists to work on commercial activities, it is actually beyond their professional ability. Besides, the tendency of journalists under the pressure of running the business weakened their investment into news production, which is detrimental to the professionalism, credibility and reputation of the media in the long run.”^6^ Thus this structural adaptation appeared not in line with the legitimacy of social benefits.

### Stable Stage: The Compartmentalizing Strategy of Hybridity in the Uncertain Institutional Environment

#### Adaptation of Hybridity

**FIGURE 2:**
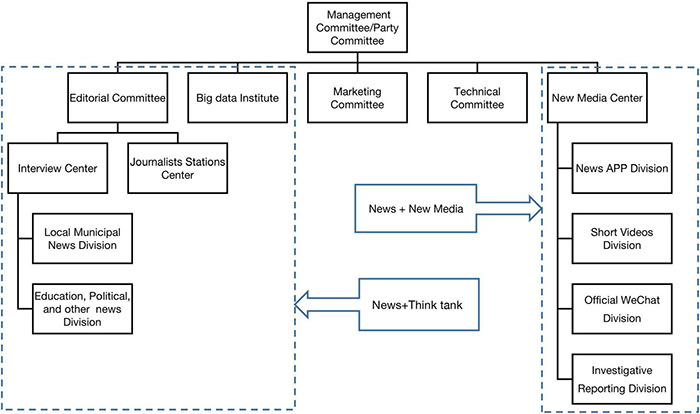
The organizational structure of N in the stable stage.

Since 2019, N had gradually abolished the “reporting command center” and the “contracted platform.” It instead started a process of reorganization based on the previous exploration which featured as deviating from the media function of public news. N’s organizational structure in this stage keeps relatively stable which is illustrated by the Figure 2.

Figure 2 shows that the new organizational structure has three additional departments: Big Data Institute, New Media Center, and Technical Committee under the Management Committee led by the Party Committee. Together with the original Editorial Committee and Marketing Committee, they form the leader board at the top level of the structure. In the manipulation, N redistributes all editors into different news divisions and implements “column responsibility system” which means every account of the official news APP and newspaper layout has been appointed specific chief editors in charge of direct operation. At the same time, the convergent editing team of the original “reporting command center” has been reorganized into News APP Division and Short videos Division which form a fresh New Media center together with the Official Wechat Division. The new Interview Center is no longer under the leadership of the editorial committee, bug undertakes the tasks of news reporting, as well as business projects of “think tank”^7^ independently. Although this organizational differentiation has greatly enhanced the autonomy of departments, N offsets the risk of organizational fracture by establishing a new “Big Data Institute” after canceling the “platforms.” It can be regarded as a symbolic institution^8^ with the main goal of managing commercial projects previously handled by the platforms. By this means, the new organizational structure constitutes an orderly flat mode under control.

When N tries to achieve diversified organizational goals through organizational differentiation, an urgent problem arises. The traditional “piece work” incentive mechanism has been incapable of suiting the functional transformation of journalists in the new media era. In the example of the new media departments, “it is difficult for the newspaper office to find the production and communication rule for audience’s attention, so there is no way to conduct performance assessment completely according to the new media data. Because many manuscripts have high news value but attract little attention online.”^9^ Moreover, after journalists turn more attention to “think tank” projects, their contribution to business results and media brand influence could not be assessed by the workload^10^.

Allowing for these tensions, N introduces the human resource management of internet companies to build a brand-new hierarchical system of professional and technical positions. All positions have transformed from the previous single position set as reporter or editor to five categories of collection-editing, research, product, R&D, and design. The personnel included in this system are paid with the “negotiated annual salary,” a fixed number established by the human resource department. Everyone’s monthly income depends on the fixed number multiplied by the percentage of the performance score. Specific assessment indicators and weights are determined by the department heads, who will set up different evaluation systems for each person according to different task assignments. The indicators include news quality, workload, communication data, social resources, projects, etc. The assessment of middle-level heads has additionally incorporated departmental coordination accounting for 40%^11^.

This new human resource management system of N has deeply increased the flexibility in journalists’ work and the management authority of middle-level heads. In fact, this aims to create more adaptive space for each task unit through the redistribution of human assets, so that they can independently explore how to better meet the requirements of the institutional environment in line with their work preference. Based on the differentiation of organizational structure, this strategy further transmits downward the legitimacy pressure to specific news departments and reporting groups.

#### Legitimacy Correlation

N’s organizational structure adaptation in the stable stage is manifested as a deeply compartmentalizing strategy. The centralized coordination mechanism of news production is replaced by a completely flat organizational structure where the decision-making power has been transferred to the middle-level structure. The department heads are responsible for controlling specific production directions, which is matched by the high dependence of human resource management on the personal judgment of leaders. Its correlation with the institutional environment is as follows.

##### Institutional Competition

For the sake of combing competing institution logics, mass media organizations entail isolating logics in different departments and divisions. For example, the New Media Center mainly takes responsibility for different technological innovations in news production (e.g., APP/Wechat/short video). The Investigative Reporting Division concentrated on high-quality news content for the legitimacy of news value. The Interview Center undertakes think-tank projects and some part of commercial goals with the Marketing Committee’s support for marketing development and operation. In these task units, the senior leaders only take charge of censorship before releasing news information, and no longer make overall arrangements for news content. Most of the management and decision-making power has been delegated to the mid-level heads which leads to a further differentiation of organizational structure.

##### Institutional Change

Continuous changes in institutional logics are more pronounced in this stage. On the one hand, after the internal integration of organizational structure featured by the “central kitchen” has been proved difficult to succeed, there never forms an explicit consensus on what is an effective reform path.

Mass media organizations must keep open to the external institutional environment as described above and shift to the deeply compartmentalizing strategy. On the other hand, the standards of different institutional logics about whether mass media is legitimate are also very ambiguous and intertwined, thereby requiring ongoing trade-offs on legitimacy.

First, the balance between news and marketing, as mentioned by a journalist:

The head requires us to do business projects along with public news, because the capacity of news production guarantees the sustainability of think tank. But the standards (of the two) are often inconsistent. For example, how to evaluate the scale, effect, and journalists’ contribution of the project is completely dependent on the personal judgment of the heads^11^.

Second, the balance between quality and distribution of news reporting. Speaking to this point, a journalist from the Investigative Reporting Division indicated: “the (department) head believes that the advantage of investigative reporting is speed. He thinks it adequate to provide additional information without excessive consideration of quality. But sometimes he criticizes us for not reaching the level of our peers and emphasizes the supervisory role of media as the safeguard of public interests.”^12^ Lastly, the balance between audience’s attention and news content. In the operation of WeChat official account, the reporter frequently finds it difficult to satisfy social expectations. “When the creative content gets huge attention, the audience censure us for lack of depth and social responsibility. But nobody cares the serious news.”^13^ In this case, it should be noted that the flexible human resource management has effectively eased and coordinated the conflicts within the organization when combining different institutional logics.

##### Integration Through Internal Legitimacy

Although the new organizational structure can create a certain innovation space for regaining legitimacy, it, in turn, leads to a strong uncertainty in the overall organizational goals. Most insiders believe that it is because “senior leaders figure out neither the direction of reform nor the way for public news, and they also aren’t familiar with the detailed tasks.”^14^ Meanwhile, after the commercial think tank projects have been charged by the Big Data Institute, the financial investment of each department is arranged through the overall budget. So the resources they can distribute are strictly controlled by the organization. Under this circumstance, departments tend to compete for resources in a fiercer manner. In the event of unclear organizational objectives, every middle-level head must strive to prove the importance and legitimacy of his department to the top echelon^15^. Within N, the legitimacy orientation centered on “online attention” and “political stance” has gradually formed^16^, as these two have the greatest certainty in China’s context. Chinese mass media organizations have spontaneously formed an organizational integration through internal legitimacy to mitigate the latent conflicts, which ultimately promoted the organic combination of the two response mechanisms of blending and compartmentalizing.

**Change 1:** To meet the needs of multiple legitimacy, mass media organizations have enhanced the horizontal differentiation of the organizational structure and the decentralization of the decision-making mechanism, creating more independent innovation space for each department.
**Change 2:** In response to the continuous changes of institutional logics, mass media organizations have established a more flexible human resource management system to coordinate the internal tensions caused by institutional uncertainty.
**Change 3:** In the absence of clear organizational goals, mass media organizations spontaneously form an integration through internal legitimacy when competing for organizational resources as a “workable certainty” connecting the unconsolidated organizational structure.
**Change 4:** In the uncertain institutional environment, mass media organizations mainly adopt compartmentalizing strategies, but achieve a certain degree of integration through internal legitimacy. That implies a convergence of two response mechanisms.

## Discussion

Over the course of this study, mass media organizations as typical hybrids have taken a variety of organizational adaptive strategies to combine different institutional logics in response to the uncertain environment. In the exploration stage, they attempted to blend and integrate all the logics represented across the organization and mitigate the internal tensions by the traditional incentive mechanism centered on the news content. However, it turned out to be not in favor of regaining legitimacy to survive because of the new conflicts that arose within the organizational structure and the changing demands of the institutional environment. In consequence, mass media organizations as described in the case shift to a deeply compartmentalizing strategy with separate units and divisions corresponding to each side in the stable stage. And a situational human resource management system has been established to reduce the conflicts. By this means, it creates a flexible structure whose malleability helps to cope with the changing institutional logics. Finally, mass media organizations generate a “workable certainty” (Luscher and Lewis, 2008) to achieve the integration which is reflected in the case as the internal legitimacy.

Sustaining hybridity in the literature has either relied on engaged organizational structures, strategies, practices, and processes to work through the conflicts (e.g., Battilana and Dorado, 2010; Battilana et al., 2015), or decided by the adaptive process that adjusts the relationship between different elements (e.g., Smith and Tushman, 2005; Jay, 2013; Dalpiaz et al., 2016). This article highlights both sides of these studies. First, the blending and compartmentalizing responses of mass media organizations resonate with the research that depicted hybrids as structurally differentiated or structurally integrated (Battilana et al., 2017; Smith and Besharov, 2019). Then the case analysis of the organizational change from the diachronic perspective reveals the provisional and negotiated response to institutional logics for navigating the ongoing tensions, which is to some extent in line with the research of adaptation (Luscher and Lewis, 2008; Jay, 2013; Smith and Besharov, 2019).

This article recurs the research implication that the institutional environment is dynamic and uncertain (Miron-Spektor et al., 2011). But this study does not follow the approach of cognitive paradoxical frames and adaptation (Smith and Lewis, 2011; Jay, 2013). It instead focuses on how the changing and competing for institutional logics during different stages affect the adaptive process of hybrids, thereby morphing into a new framework. In this sense, the article adds some institutional nuance on account of sustaining organizational hybridity by re-conceptualizing “uncertainty” with “institutional competition” and “institutional change.” This research enriches the discussions on organizations’ managerial strategic choices influenced by the evolution of the institutional logics from the legitimation-as-process perspective (Thelisson et al., 2018; Thelisson and Meier, 2020).

Additionally, this article expands the existing research from two dimensions. First, there form two kinds of managerial strategic choices in response to the nature of institutional change. On the one hand, mass media organizations shift from the dominating blending strategy to the deeply compartmentalizing strategy based on different demands of competing for institutional logics in different stages. On the other hand, they adopt a flexible organizational structure to handle the goal ambiguity, which can be seen as a further decentralization of hybridity. Secondly, the case evolves an organizational integration based on internal legitimacy spontaneously. It manifests a possibility for hybrids of combining the two major response mechanisms in one process. Therefore, these findings contribute to move beyond the literature which depicts hybrids as either differentiated or integrated, and treat them as static (Smith and Besharov, 2019).

## Concluding Remarks

This research has found that under the condition of extreme uncertainties in technology and market, mass media organizations attempt to improve their viability by obtaining external legitimacy. But the complicated environment is increasing the complexity of this process. It has become the core issue in the discussions over hybridity that how mass media organizations respond to the uncertain institutional logics featured by mutual competition and continuous change. Chinese mass media organizations provide a possible answer—to cope with different legitimacy pressures by enhancing the uncertainty of organizational structure within a controllable range.

This uncertainty includes two main dimensions: organizational objectives and organizational incentive mechanism. In this case study, the early “central kitchen” model is a structural adaptation aimed at “news content,” expanding the organizational flexibility through project systems and contracted platforms. Confronting the new tensions inside and outside, this organization takes a compartmentalizing strategy and enhances the uncertainty of organizational structure further at two levels. This change consequently decomposes the legitimacy pressure from top to down by improving organizational differentiation and strengthening departmental independence, thereby constructing a relatively open and independent exploration model. However, the uncertainty of institutional logics also increases the vagueness of organizational objectives, resulting in the instability and contradiction of the internal incentive mechanism. The final organizational structure returns to a bottom-up integration through internal legitimacy and achieves the internalization of external institutional pressures. This is a China’s unique media practical experience.

From a theoretical point of view, the importance of this case study is to provide a most typical template to explain how hybrid organizations form an endogenous structural balance in a complex institutional environment without obvious market bias and signals. Obviously, the combination of the blending and compartmentalizing strategies will determine the distribution of uncertainties within the organization and eventually shape the form of hybridity, and vice versa. Future research could further explore the influence mechanism and formation logic of organizational structure change. For example, under what circumstances will there generate an integration or differentiation, which mechanisms are functioning, and so on. Meanwhile, these theoretical discussions will help a better understanding of the structural change of hybrids.

The empirical data has shown that in the general crisis of journalism, organizational differentiation may be an inevitable path for mass media organizations to deal with the challenges posed by institutional logics. Even in a relatively open organizational structure, how to distribute and regulate the uncertainties of different dimensions will greatly affect news production and public life. For example, the media organization, in this case, has encountered a serious contradiction between organizational legitimacy and efficiency. Due to the lack of normalized news coordination mechanism, not only have much important news been not well presented, but the fragmented operation by departments has led to many ineffective competitions. Therefore, how to deal with the degree and scope of uncertainties will become an important problem that mass media in the future should consider. However, even though this article responds to calls for research in contexts other than North America (Greenwood et al., 2010), questions about whether these findings are unique to Chinese mass media organizations or commonly in other organization field still need further comparative studies.

## Data Availability Statement

The raw data supporting the conclusions of this article will be made available by the authors, without undue reservation.

## Author Contributions

Both authors listed have made a substantial, direct, and intellectual contribution to the work, and approved it for publication.

## Conflict of Interest

The authors declare that the research was conducted in the absence of any commercial or financial relationships that could be construed as a potential conflict of interest.

## Publisher’s Note

All claims expressed in this article are solely those of the authors and do not necessarily represent those of their affiliated organizations, or those of the publisher, the editors and the reviewers. Any product that may be evaluated in this article, or claim that may be made by its manufacturer, is not guaranteed or endorsed by the publisher.

## Footnotes

1 The term “hybridity” is used in two situations in the literature. One is “neither market nor hierarchy,” but a hybrid, networked organizational form (Powell, 1990). The other refers to organizations combing different logics such as public and private organizational goals (Jay, 2013). This article uses it in the latter sense.

2 Authors’ interview with one editor conducted on August 21, 2017.

3 Authors’ interview with one journalist of the investigative reporting division conducted on September 30, 2017.

4 Authors’ interview with one editor conducted on September 25, 2017.

5 Authors’ interview with one editor conducted September 12, 2017.

6 Author’s interview with one mid-level head conducted December 23, 2020.

7 In 2018, N began an organizational transformation from news media to media think tank which means developing some research projects to serve the enterprises and government as a new business model.

8 Because in addition to a small number of principals and technicians, the main staff of this department are concurrently held by journalists from the Interview Center.

9 Authors’ interview with one mid-level head conducted on September 28, 2017.

10 Authors’ interview with one mid-level head conducted on July 14, 2021.

11 Authors’ interview with one journalist from the interview center conducted on December 20, 2021.

12 Authors’ interview with one journalist from the investigating reporting division conducted on December 1, 2021.

13 Authors’ interview with one reporter from the official weChat division conducted on December 4, 2020.

14 Authors’ interviews with two mid-level heads conducted on July 14, 2021 and 23 December 23, 2020.

15 Authors’ interviews with three insiders conducted on November 27, December 4, 2020, and July 14, 2021.

16 Authors’ interviews with some insiders from November 2020 to December 2021.
